# Sex differences in corneal neovascularization in response to superficial corneal cautery in the rat

**DOI:** 10.1371/journal.pone.0221566

**Published:** 2019-09-03

**Authors:** Yazad D. Irani, Emily Pulford, Lauren Mortimer, Swati Irani, Lisa Butler, Sonja Klebe, Keryn A. Williams

**Affiliations:** 1 Discipline of Ophthalmology, College of Medicine and Public Health, Flinders University, Adelaide, Australia; 2 Discipline of Anatomical Pathology, College of Medicine and Public Health, Flinders University, Adelaide, Australia; 3 Freemasons Foundation Centre for Men's Health, Adelaide Medical School, University of Adelaide, Adelaide, Australia; 4 South Australian Health and Medical Research Institute, Adelaide, Australia; University of Florida, UNITED STATES

## Abstract

Sex-based differences in susceptibility have been reported for a number of neovascular ocular diseases. We quantified corneal neovascularization, induced by superficial silver nitrate cautery, in male and female inbred albino Sprague-Dawley, inbred albino Fischer 344, outbred pigmented Hooded Wistar and inbred pigmented Dark Agouti rats of a range of ages. Corneal neovascular area was quantified on haematoxylin-stained corneal flatmounts by image analysis. Pro-and anti-angiogenic gene expression was measured early in the neovascular response by quantitative real-time polymerase chain reaction. Androgen and estrogen receptor expression was assessed by immunohistochemistry. Male rats from all strains, with or without ocular pigmentation, exhibited significantly greater corneal neovascular area than females: Sprague-Dawley males 43±12% (n = 8), females 25±5% (n = 12), p = 0.001; Fischer 344 males 38±10% (n = 12) females 27±8% (n = 8) p = 0.043; Hooded Wistar males 32±6% (n = 8) females 22±5% (n = 12) p = 0.002; Dark Agouti males 37±11% (n = 9) females 26±7% (n = 9) p = 0.015. Corneal vascular endothelial cells expressed neither androgen nor estrogen receptor. The expression in cornea post-cautery of *Cox-2*, *Vegf-a* and *Vegf-r2* was significantly higher in males compared with females and *Vegf-r1* was significantly lower in the cornea of males compared to females, p<0.001 for each comparison. These data suggest that male corneas are primed for angiogenesis through a signalling nexus involving *Cox-2*, *Vegf-a*, and *Vegf* receptors 1 and 2. Our findings re-enforce that pre-clinical animal models of human diseases should account for sex-based differences in their design and highlight the need for well characterized and reproducible pre-clinical studies that include both male and female animals.

## Introduction

Sex-based differences in incidence and/or prevalence have been observed in a number of ocular diseases [1–5]. In particular, those characterized by the aberrant growth of new blood vessels, such as diabetic retinopathy and age-related macular degeneration, have been shown to display a sex bias [6, 7]. The reasons for this bias remain unclear.

Under normal circumstances, the cornea is avascular despite the presence of pro-angiogenic molecules including VEGF-A [8], cyclooxygenase 2 (COX-2) [9], insulin-like growth factor, fibroblast growth factor and matrix metalloproteinases [10]. Corneal avascularity is in part maintained by the expression of soluble VEGF receptor-1 which sequesters free VEGF-A, preventing it from binding to VEGF receptor-2 and mediating its pro-angiogenic effect [11]. A complex interaction and tightly regulated balance between pro- and anti-angiogenic factors maintains blood vessels at the corneo-scleral junction (the limbus) in a quiescent state. Injury, infection or inflammation can result in aberrant growth of blood vessels into the cornea [12]. The expression of VEGF-A has been shown to be increased both in humans with corneal neovascularisation [8, 13] and in animal models of corneal neovascularization [14]. The presence of abnormal corneal blood vessels is not only potentially sight threatening but is also an independent risk factor for the failure of a corneal graft [15].

We recently reported that neutralization of VEGF-B using an antibody fragment resulted in regression of corneal blood vessels in a rat model of corneal neovascularization [16]. During the course of these studies, we observed a high degree of variability in the neovascular response in the control group, in which corneas were cauterised but otherwise untreated. We re-analyzed the data after separating by sex. We found that although there was still variation within males and females, there was a significant difference in neovascularization between male and female rats, with male rats consistently demonstrating more neovascularization than females. Previous work in our laboratory had demonstrated that susceptibility to oxygen-induced retinopathy, another ocular neovascular disease, was affected by rat strain and ocular pigmentation [17]. These data taken together prompted us to design the current study in which we investigated whether the sex-based difference in corneal neovascularization observed in adult albino Sprague Dawley rats was peculiar to this strain, or was generalizable to rats of other strains, ocular pigmentation, and age. We used albino (Fischer 344, Sprague Dawley) as well as pigmented (Hooded Wistar, Dark Agouti) strains.

We sought to uncover the mechanism of the observed sex difference by quantifying the expression of genes known to be involved in angiogenesis using quantitative reverse transcriptase polymerase chain reaction (qRT-PCR). The expression of genes known to affect the ocular vasculature such as *Ang-2*, *Cox-2*, *Igf-1*, *Nk1r*, *Pedf*, *Tie2*, *Vegf-a* and their receptors [18, 19], was quantified. A fundamental difference between males and females is the expression of steroid sex hormones and their receptors. Furthermore, sex hormones and their receptors have been shown to exert an effect on angiogenesis, and mRNAs for testosterone, oestrogen and progesterone receptors have been found in the eyes of rabbits and humans [20], including in the human cornea [21]. We investigated the expression of the estrogen receptor and the androgen receptor in the cornea to elucidate if they were differentially expressed in male and female rats under normal conditions as well as post-cautery.

There is a growing call from the scientific community to improve the quality of pre-clinical testing before progression to clinical trials. The failure of novel therapies in clinical trials, after promising pre-clinical results, could be influenced by inadequate sex balance in pre-clinical studies. Indeed, the majority of pre-clinical testing in biomedical fields has a bias towards the use of male animals [22]. Recognizing this issue, the National Institute of Health has enacted policy for the inclusion of sex in pre-clinical experimental design [23]. This study reports a sex-related difference in corneal neovascularization and adds weight to the push for the inclusion of both male and female animals in pre-clinical models of human disease.

## Results

### Differences in the degree of corneal neovascularization between male and female rats

**Fig 1:**
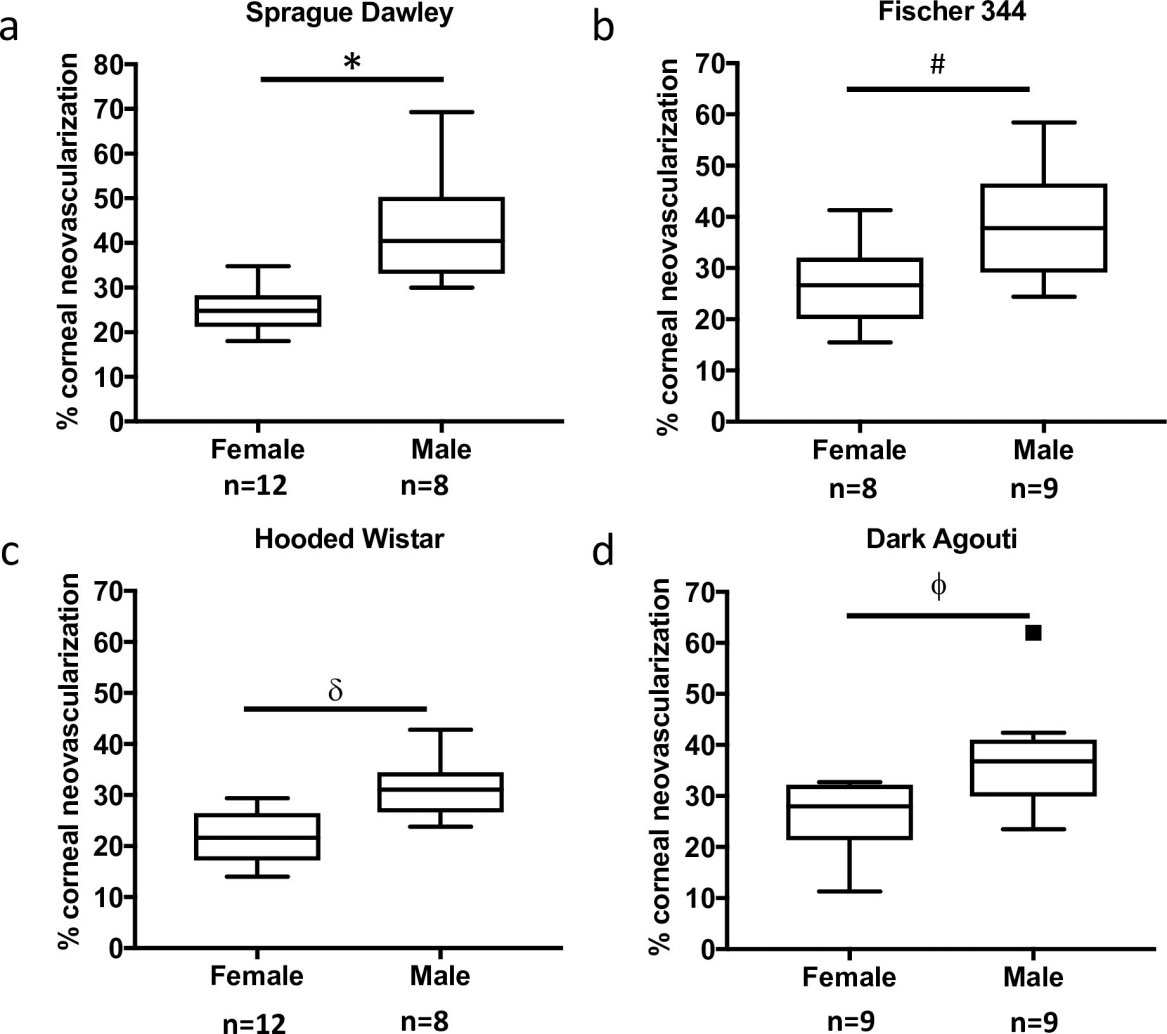
Sex differences in corneal neovascularization are independent of strain or pigmentation. The degree of corneal neovascularization 14 days post-silver nitrate cautery was compared in male and female albino (a) Sprague-Dawley (12 female and 8 male), (b) Fischer 344 (8 females and 9 males); or pigmented, (c) Hooded Wistar (12 females and 8 males, pigmented eyes), (d) Dark Agouti (9 females and 9 males), rat strains. Male rats developed significantly more vessels compared with females, regardless of strain (Sprague-Dawley *p = 0.001, Fischer 344 ^#^p = 0.043, Hooded Wistar ^δ^p = 0.002, Dark Agouti ^ϕ^p = 0.015). The box covers data within quartiles one and three, with the median represented by the line through the box. The whiskers represent the spread of the data within 50% of quartile one and quartile three; outliers are represented by squares.

Corneal neovascularization was induced in female and male rats by superficial cautery (S1 Fig). Oedema and neo-vessels were evident in both female and male rats up to 7 days post-cautery (S1 Fig). The oedema had resolved by day 14 in both sexes (S1 Fig). The percentage of the cornea covered by blood vessels was quantified in male and female Sprague-Dawley, Fischer 344, Hooded Wistar, and Dark Agouti rats at 14 days post-cautery (Fig 1A–1D). The albino Sprague-Dawley and Fischer 344 strains (Fig 1A and 1B) as well as the pigmented Hooded Wistar and Dark Agouti strains (Fig 1C and 1D) all demonstrated a sex-based difference in corneal neovascularization. Since the differences in corneal neovascularization were independent of strain, further experiments were carried out using Sprague Dawley rats, which showed the greatest difference between males and females (72% more vessels in males than females, compared with 42% in Fischer 344, 45% in Hooded Wistar and 42% in Dark Agouti). Representative flatmounts of haematoxylin perfused corneal neovasculatures are shown in S2 Fig.

### Histology

**Fig 2:**
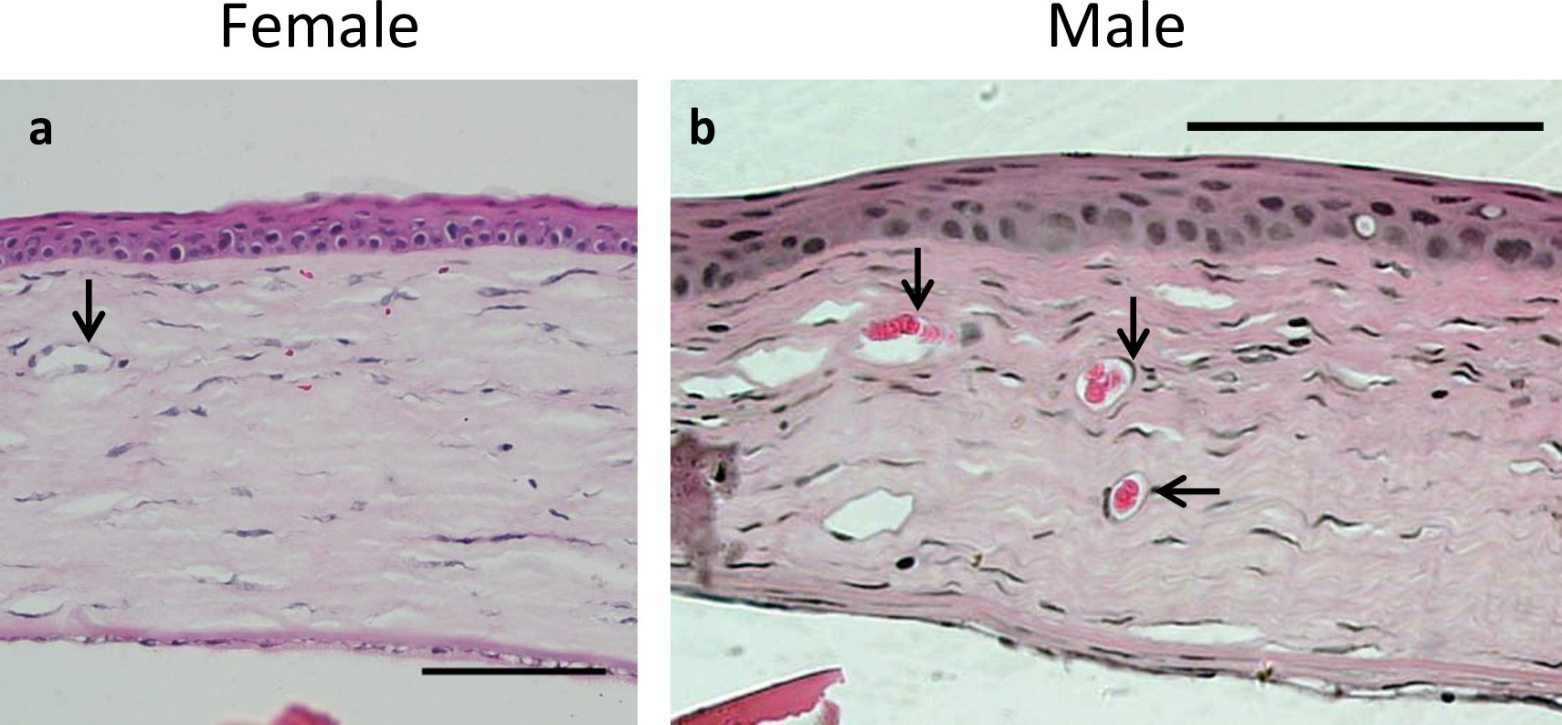
Histological analysis showed patent blood vessels in both male and female rats 14 days post-corneal cautery. Corneal sections from both **(a)** female and **(b)** male corneas exhibited near-normal histology 14 days post-cautery. The epithelium and endothelium were normal in appearance. Patent blood vessels were apparent (black arrows) in both females and males. Scale bars 100 μm.

Corneal sections from male and female Sprague-Dawley rats were examined by haematoxylin and eosin staining 14 days post-cautery. Corneal sections demonstrated near-normal structure. The corneal epithelium was continuous and consisted of 4–5 layers of cells. Some oedema was apparent in the corneal stroma and patent blood vessels were observed in both females and males (Fig 2A and 2B). Descemet’s membrane appeared normal with an intact monolayer of corneal endothelial cells. Masked, semi-quantitative assessment of corneas of male and female rats indicated that the corneal inflammatory cell infiltrate was variable, but there was no clear sex-related difference observed in the extent of inflammation at 72 hours or 14 days post cautery (S1 Table).

### Immunohistochemistry for sex hormone receptors

**Fig 3:**
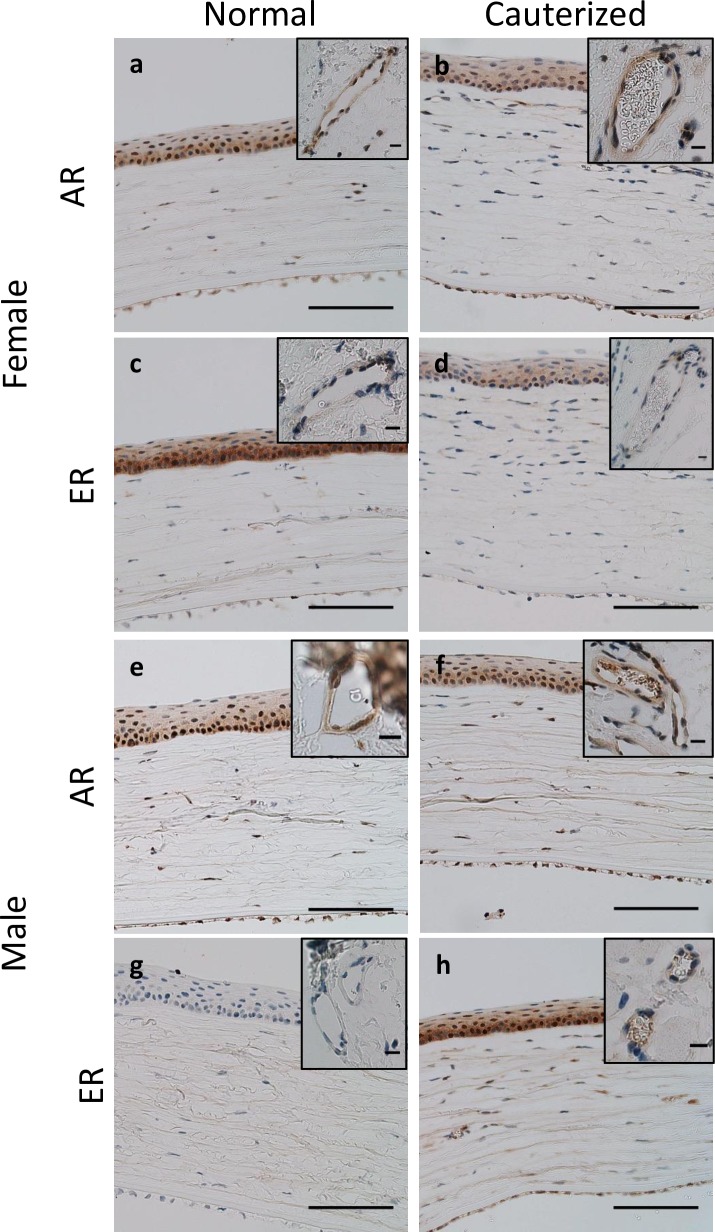
Immunohistochemistry for androgen receptor and estrogen receptor in normal and cauterized corneas. **(a, c, g, e)** Normal and **(b, d, f, h)** cauterized corneal sections (72 h post-cautery) labelled with **(a, b, e, f)** androgen receptor, and **(c, d, g, h)** estrogen receptor antibody. Panels **a-d** show corneas from female rats while panels **e-h** show corneas from male rats. Inserts depict labelling on blood vessels in the cornea of cauterized eyes and at the limbal arcades of non-cauterized eyes. Positive nuclear labelling for androgen receptor was observed in both female and male untreated corneas as well as cauterized corneas. The nuclei of vascular endothelial cells were negative. Estrogen receptor was expressed in the nuclei of untreated female corneas but was not observed in untreated male corneas. Expression of estrogen receptor was observed in the corneal epithelium of male rats after corneal cautery. The nuclei of blood vessel endothelial cells were negative for estrogen receptor. Scale bars 100 μm, insert 10 μm.

The expression of the androgen receptor and estrogen receptor was assessed by immunohistochemistry in female and male untreated rats and treated rats at 72 h after corneal cautery. Blood vessel endothelial cells expressed neither the androgen nor the estrogen receptor (Fig 3A–3H). The androgen receptor was expressed in the corneal epithelium, indicated by nuclear labelling, in females and males under normal conditions and after cautery (Fig 3A, 3B, 3E and 3F). The estrogen receptor was expressed in a nuclear distribution in the corneal epithelium of female rats under normal conditions and post-cautery (Fig 3C and 3D). Male rats did not express estrogen receptor under normal conditions, however, positive expression was observed in the corneal epithelium post-cautery (Fig 3G and 3H).

### Differential gene expression in male and female rats in response to corneal cautery

Differential expression of genes known to be involved in angiogenesis was examined by qRT-PCR. Tissue was collected at 72 h post-cautery (at which point neovascular buds were generally apparent at the limbal arcades), so as to examine genes involved in the initiation and early stages of neovascularization. A difference in gene expression in response to cautery was detected for all the genes tested, however, mRNAs for *Cox-2*, *Vegf-a*, *Vegf-r1*, *Vegf-r2 and Vegf-b* also displayed a quantitative sex-based difference.

**Fig 4:**
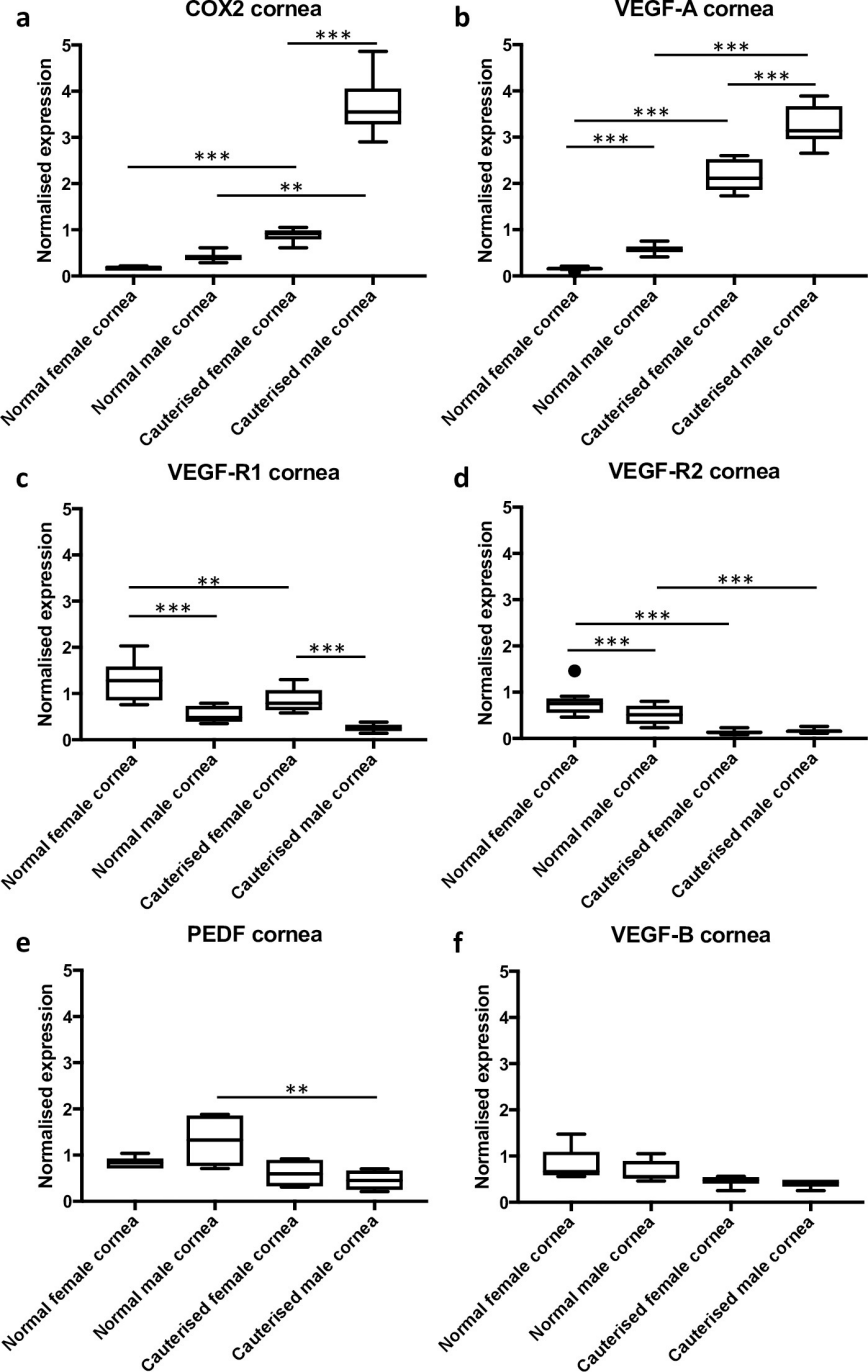
Differential gene expression in the corneas of male and female rats in response to cautery. The expression of genes in the cornea was examined 72 h post-cautery. (a) *Cox-2* mRNA expression was significantly upregulated in both males and females, however males expressed significantly more Cox-2 in response to cautery. (b) *Vegf-a* was upregulated in both males and females post-cautery. (c) *Vegf-r1* expression was significantly lower in males compared to females post-cautery. (d) *Vegf-r2* was downregulated in both males and females post-cautery. (e) *Pedf* expression was downregulated post-cautery in males but not in females. (f) There was no significant difference in the expression of *Vegf-*b. The y-axis depicts relative gene expression, normalized to the geometric mean of two reference genes. The data are represented as box and whisker plots with the box depicting the middle two quartiles and the whiskers the spread of data; outliers are marked by a circle, ** p<0.01, *** p<0.001.

The expression of *Cox-2* mRNA was significantly increased in male cauterised corneas compared with normal male corneas and female cauterised corneas (Fig 4A). *Vegf-a* mRNA, encoding a key regulator of angiogenesis, was upregulated in the corneas of both male and female rats in response to cautery, however male cauterised corneas expressed significantly more *Vegf-a* mRNA than female cauterised corneas (Fig 4B). *Vegf-r1* mRNA was downregulated in female corneas upon cautery and there was no significant difference in expression between normal and cauterised male corneas, however normal male corneas exhibited significantly lower levels of *Vegf-r1* mRNA compared with normal female corneas (Fig 4C). We assessed VEGF-R1 protein expression in corneas by immunohistochemistry. We demonstrated that female corneas had greater VEGF-R1 expression compared with males (S3 Fig). These data were in concordance with gene expression data. Expression was observed mainly around the corneal endothelium and was associated with an inflammatory infiltrate. *Vegf-r2* mRNA was significantly downregulated in males and females upon cautery (Fig 4D). *Pedf* mRNA, which encodes an anti-angiogenic protein, was downregulated in males upon cautery but remained unchanged in females (Fig 4E). The expression of *Vegf-b* mRNA in the cornea was unaffected by cautery (Fig 4F).

**Fig 5:**
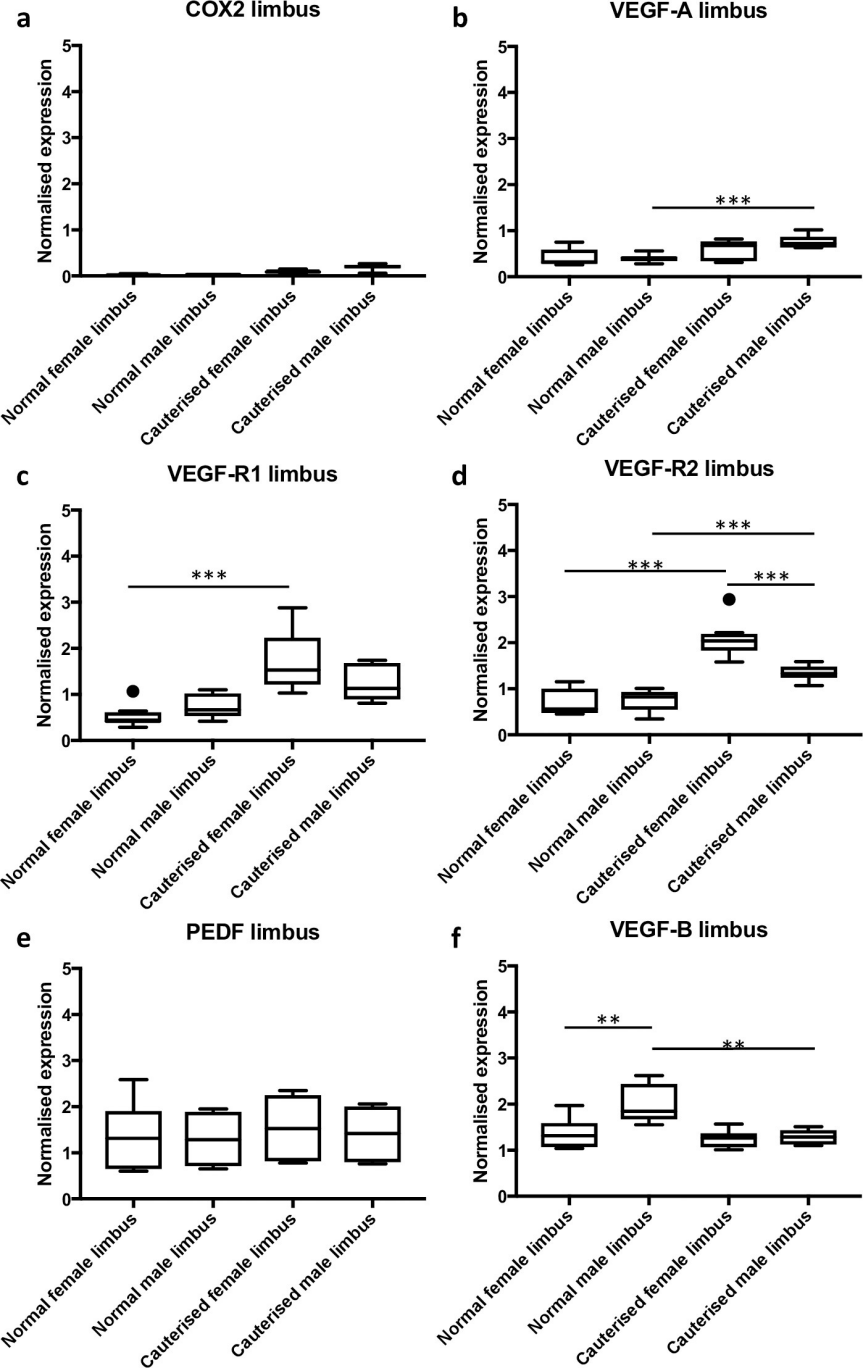
Differential gene expression in the limbus of male and female rats in response to cautery. The expression of angiogenesis genes in the limbus was examined at 72 h post-cautery. (a) *Cox-2* was not detected. (b) *Vegf-a* was upregulated in males but not females post-cautery. (c) *Vegf-r1* expression was significantly higher in cauterized female limbus when compared to normal female limbus. (d) *Vegf-r2* was upregulated in both males and females following cautery. (e) There were no significant differences in the expression of *Pedf* (f) *Vegf-b* expression was higher in males compared to females under normal circumstances and was significantly reduced in males post-cautery. The y-axis depicts relative gene expression, normalized to the geometric mean of two reference genes. The data are represented as box and whisker plots with the box depicting the middle two quartiles and the whiskers the spread of data; outliers are marked by a circle, ** p<0.01, *** p<0.001.

In the limbus, *Cox-2* mRNA was not detected (Fig 5A), *Vegf-a* mRNA was significantly upregulated in male normal compared with cauterised corneas (Fig 5B). *Vegf-r1* mRNA was significantly upregulated in the limbus of females upon cautery (Fig 5C). Cautery resulted in upregulation of *Vegf-r2* mRNA in the limbus of both males and females (Fig 5D). Limbal expression of *Pedf* mRNA was not significantly different in males or females upon cautery (Fig 5E). *Vegf-b* mRNA was significantly downregulated in males post-cautery and was not significantly changed in females (Fig 5F). A summary of the normalized expression of all the genes tested is provided in S2 Table.

## Discussion

Our study demonstrated a significant sex-related difference in the neovascular response to superficial chemical cautery of the cornea in a rat model. The sex difference was observed in 4 strains of rats, including inbred and outbred strains, and strains with or without ocular pigmentation, and when animals were stratified by age (young to old). The robust, statistically significant sex difference was observed over and above intrinsic biological variation within each group. Furthermore, we observed differential expression of genes involved in the regulation of angiogenesis between male and female rats.

Large population studies in Caucasians [24–26] have shown that women are at a moderately higher risk of developing age-related macular degeneration, while the converse is true in Asian populations [27, 28]. The mechanisms of the observed differences have not been elucidated. However, exposure to exogenous oestrogen has been associated with a reduced risk of developing age-related macular degeneration [29, 30]. Similarly, the data surrounding sex differences in diabetic retinopathy are mixed. Increased levels of oestrogen and progesterone during pregnancy have been associated with the progression of diabetic retinopathy [31, 32]. However, in men, high testosterone levels are associated with an increased risk of developing diabetic retinopathy [33, 34]. Taken together the above data suggest a possible role of the steroid sex hormones and their receptors in ocular neovascular disease. Testosterone and oestrogen have been shown to have pro-angiogenic [35–39] as well as anti-angiogenic effects in rodent models [40–43]. We observed expression of the androgen receptor and the oestrogen receptor in the corneal epithelium, but sex hormone receptors were not detected on vascular endothelial cells, suggesting that the sex hormones did not play a direct role in ocular angiogenesis by acting on vascular endothelial cells in our model. However, a paracrine effect mediated by corneal epithelial cells cannot be discounted.

We quantified the expression of genes involved in the regulation of angiogenesis and inflammation, in a bid to uncover the mechanisms behind the observed sex-difference in corneal neovascularization. The 72-hour timepoint was chosen as neovascular buds were apparent at the limbal arcades between 3–4 days post-cautery, and we aimed to capture the genes that drive the neovascular response. Upon cautery, we observed in increase in mRNAs for *Vegf-a* in the cornea and its receptor *Vegf-r2* in the limbus in both males and females. These results were expected, as the interaction of VEGF-A and VEGF-R2 is a key driver of angiogenesis. We were however, interested in teasing out sex-related differential gene expression in response to cautery.

The gene that showed the greatest change in expression between females and males upon cautery was *Cox-2* (Fig 4A). *Cox-2* is a key enzyme in inflammatory cytokine-induced angiogenesis [44]. We employed the silver nitrate cautery model of corneal angiogenesis, a clinically relevant model, which induces inflammation as well as neovascularization [45]. The importance of considering the sex of experimental animals in preclinical research on inflammatory diseases has recently been reviewed [46]. The extent of inflammation can vary between males and females. One report describing rodent models of acute inflammation indicated that COX-2 expression and prostaglandin levels produced by neutrophil infiltration into inflamed tissue were higher in male than female animals [47]. In a rat model of traumatic brain injury, COX-2 mRNA and protein levels were significantly higher in the perilesional areas of males at 24 hours and 72 hours, the time of peak histological inflammation, than in their female counterparts [48]. We noted that male rat corneas appeared more inflamed and oedematous following cautery when compared to female rats (S1 Fig). *Cox-2* inhibition by RNAi has been shown to reduce the protein levels of key angiogenesis regulators, VEGF-A, MMP2 and MMP9 *in vitro* [49]. Pharmacological inhibition of COX-2 with small molecule inhibitors also inhibits angiogenesis, possibly through the MAP kinase pathway [50]. We have previously shown differential mRNA expression of *Cox-2* in rat strains that are susceptible and resistant to oxygen-induced retinopathy in a rat model [18]. These data suggest that *Cox-2* over-expression in males could potentiate increased angiogenesis through its effects on *Vegf-a*, which was significantly overexpressed in the cornea of males compared to females following cautery (Fig 4A). COX-2 might thus be a potential target for anti-angiogenic therapy in corneal neovascularization in humans. Of relevance, the selective COX-2 inhibitor Celecoxib has been shown to reduce tumour angiogenesis [51]. Furthermore, non-steroidal anti-inflammatory agents and a selective COX-2 inhibitor have been shown to partially inhibit corneal angiogenesis in a mouse model [52]. Testing the anti-angiogenic effects of clinically approved anti-human VEGF-A antibodies such as bevacizumab and ranibizumab in rodent models is not possible as these agents do not bind effectively to either mouse or rat VEGF-A [53]. Inhibition of VEGF-A or COX-2 individually, partially inhibits but does not prevent corneal neovascularization [52, 54]. However, we speculate that dual inhibition of VEGF-A and COX-2 might potentially synergize, resulting in a greater anti-angiogenic effect.

The expression of *Vegf-r1* was significantly higher in normal female than normal male corneas. This difference was maintained post-cautery, with male corneas expressing significantly less *Vegf-r1* than female corneas (Fig 4C). Interestingly, in the limbus there was a significant increase in *Vegf-r1*expression in response to cautery in females only. Soluble VEGF-R1 has been shown to be responsible for the maintenance of corneal avascularity in mice [11] as well as humans [55]. VEGF-B, which is a ligand for VEGF-R1, showed significantly higher expression (mRNA) in normal male limbus compared with normal female limbus, and such expression decreased significantly after cautery in males only. VEGF-B is thought to be dispensable for the growth of new blood vessels but is required for their survival [56], and we have previously shown that neutralization of VEGF-B leads to regression of established (but not new) blood vessels in the rat cornea [16]. The interaction of VEGF-B with cell-bound and soluble receptor may play a role in neovascularization, however, it is likely that the interaction of VEGF-A protein with VEGF-R2 was the main driver of corneal angiogenesis in our model. *Pedf*, which is anti-angiogenic, was downregulated in male corneas upon cautery. PEDF has been shown to inhibit VEGF-A induced angiogenesis [57] in the eye [58]. Anti-angiogenic factors have been shown to play an important role in the regulation of angiogenesis and may be just as important as pro-inflammatory and pro-angiogenic factors in the observed sex differences.

A complex interaction and tightly regulated balance between pro- and anti-angiogenic factors is responsible for maintaining corneal avascularity. The introduction of angiogenic stimulus skew this balance towards the pro-angiogeneic factors. Our findings suggest that an increase in *Cox-2*, *Vegf-a*, *Vegf-r2* and a decrease in *Vegf-r1* expression in males, compared with females, might mediate increased corneal neovascularization in response to corneal cautery.

Pre-clinical animal models are often biased towards the use of males [22]. Recognising this issue, the National Institutes of Health in the United States of America now recommends the use of both male and female animals and cells in pre-clinical models [59]. The authors point out that the use of a single sex in animal models might be to blame for the failure of a number of promising preclinical results to be successfully translated to phase I/II clinical trials. Furthermore, women have been shown to experience higher rates of adverse drug reactions than men [60]. There is a growing consensus that the standards of pre-clinical testing of novel therapies should mirror clinical trials [61], with an emphasis on well characterized [62], reproducible [23] models, and with the same transparency in reporting [63, 64]. Our study employed an extensively characterized model of corneal neovascularization, male and female experimental animals of different genetic backgrounds, and of varying ages from young to old. Furthermore, our analyses were carried out by observers masked to the strains and experimental groups. Our findings reinforce the need to account for sex-based differences in the design of pre-clinical models.

## Materials and methods

### Rats

A total of 81 rats were used in this work. Sprague-Dawley (outbred albino, 12 female and 8 male for measurement of cautery and 3 female and 3 male for gene expression), Fischer 344 (inbred albino, 8 female and 9 male), Hooded Wistar (outbred pigmented, 12 female and 8 male) and Dark Agouti (inbred pigmented, 9 female and 9 male) rats, which represent inbred, outbred, albino and pigmented strains, were sourced from and housed in the Flinders University Animal Facility and exposed to a 12-hour light-dark cycle. Animals ranged in age from 12–60 weeks at the time of induction of corneal neovascularization. All animal experiments were approved by the Animal Welfare Committee of the Flinders University of South Australia and were in accordance with the ARVO Statement for the Use of Animals in Ophthalmic and Vision Research, as well as the Australian Code of Practice for the Care and Use of Animals for Scientific Purposes. All procedures were performed under general anaesthesia. In addition, topical anaesthetic eye drops (proxymetacaine hydrochloride, 0.5% w/v, Bausch & Lomb, Kingston-Upon-Thames, Surrey, UK) were used prior to corneal cautery and the rats were administered paracetamol (0.5 mg/ml, Panadol, GlaxoSmithKline, Brentford, United Kingdom) in the drinking water following cautery. Images were captured using an Olympus E-330 digital camera attached to a Leica Wild M690 ophthalmic surgical microscope.

### Induction and quantification of corneal neovascularization in rats

Corneal neovascularization was induced and quantified as previously described [16]. Briefly, corneal neovascularization was induced by superficial cautery using a silver nitrate potassium nitrate applicator (Grafco, QLD, Australia). This is a clinically relevant model that induces both inflammation and neovascularization. Following euthanasia, corneal neovessels were perfused with haematoxylin, and the corneas were then flatmounted and imaged. The images were coded and the corneal neovascular area was quantified using ImageJ software (NIH, Bethesda, MD) by an observer masked to the strain or treatment group (untreated, cauterised), as previously described [16]. Briefly, RGB images of corneal flat mounts were split on to the individual channels. The green channel was used for further analysis as it provided maximum contrast. The threshold function was applied to remove background and the area of neovascularization was calculated using the analyze particles function.

### Histology and immunohistochemistry

Formalin-fixed paraffin embedded tissue was sectioned at 6 μm. The sections were deparaffinised in xylene and rehydrated through graded alcohol. Sections were either stained with haematoxylin and eosin or processed for immunohistochemistry. H&E stained corneal sections at 72 hours and day 14 post-cautery were assessed by a pathologist, who was masked to the sex of the animal from which the sample was derived. Semi-quantitative scoring of the inflammatory infiltrate present within the cornea (excluding hypopyon) was performed. For immnohistochemical analysis, endogenous peroxidase activity was quenched with 3% hydrogen peroxide. Sections were then incubated with estrogen receptor antibody (1:200, NCL-L-ER-6F11, Leica Biosystems, Wetzlar, Germany), androgen receptor antibody (1:100, N-20 sc-816, Santa Cruz Biotechnology, Dallas, TX, USA) or VEGF-R1 antibody (1:100, ab32152, Abcam, Cambridge, United Kingdom) overnight. Detection was performed using the NovoLink polymer detection system (Novocastra Laboratories, Newcastle Upon Tyne, UK) according to the manufacturer’s instructions. Nuclear labelling was considered specific for androgen and estrogen receptor, while cytoplasmic labelling was considered specific for VEGF-R1.

### Gene expression by quantitative reverse transcriptase PCR

The corneas of male and female Sprague Dawley rats (n = 3 for each group) were cauterised as described above. Seventy-two hours post-cautery rats were euthanized, and the eyes harvested, immediately post-mortem. This timepoint was chosen as neovascular buds were apparent at the limbal arcades 3–4 days post cautery and we aimed to measure genes that were driving the inflammation and corneal neovascularization. The eyes were dissected immediately in ice. Corneal buttons and limbo-scleral rings were dissected and snap frozen in liquid nitrogen. RNA was extracted using Triazol reagent and reverse transcribed (using Superscript III reverse transcriptase, Thermo Fischer Scientific) as described elsewhere [65]. Two-step quantitative reverse transcriptase PCR was performed to measure the expression of genes previously described as involved with inflammation and angiogenesis in the eye, such as angiopoietin-2, cyclooxygenase-2, insulin like growth factor 1, neurokinin 1 receptor, pigment epithelium derived factor, TEK receptor tyrosine kinase 2, vascular endothelial growth factor A, vascular endothelial growth factor B, vascular endothelial growth factor receptor 1, and vascular endothelial growth factor receptor 2 as described elsewhere [18]. Briefly, cDNA and primers were combined with PowerUp SYBR Green master mix (ThermoFischer Scientific, Waltham, MA, USA) according to the manufacturer’s instructions. Amplification was carried out using a StepOnePlus Real-Time PCR system (ThermoFischer Scientific), with initial denaturation at 95°C for 10 min, followed by 40 cycles of denaturation at 95°C for 1 min, annealing at 60°C for 30 seconds and amplification at 72°C for 1 min. Melt curve analysis was performed at 1°C intervals from 55°C to 95°C. The 2^–ΔΔ**Ct**^ method was used to quantify gene expression with expression normalised to the geometric means of two reference genes (β-actin and hypoxanthine guanine phosphoribosyl transferase). The primers used to amplify genes of interest are described in Table 1 and standard curves for calculation of amplification efficiency are displayed in S4 Fig.

**Table 1:** Primer sequences for quantitative reverse transcriptase polymerase chain reaction.

Gene	Primer sequence 5`-3`	Tm (°C)	Amplicon size (base pairs)
Angiopoietin 2	For—CAGCTTGCTGACCATGATGT	60	87
	Rev—GCACAGTCTCTGAAGGTGGTT	59	
β-Actin	For—CCTCTGAACCCTAAGGCCAACC	62	94
	Rev—ACACAGCCTGGATGGCTACG	62	
Cyclooxygenase 2	For—TCCTCCTTGAACACGGACTT	59	102
	Rev—CTGCTTGTACAGCGATTGGA	60	
Hypoxanthine guanine phosphoribosyl transferase	For—TTGTTGGATATGCCCTTGACT	60	104
	Rev—CCGCTGTCTTTTAGGCTTTG	59	
Insulin-like growth factor 1	For—CACACTGACATGCCCAAGA	59	105
	Rev—GGGAGGCTCCTCCTACTGAC	60	
Neurokinin 1 receptor	For—GTTGCCTCAACGACAGGTTC	59	117
	Rev—GTGTCTGGAGGTATCGGGTG	60	
Pigment epithelium derived factor	For—CAGCCAGAATGTCCCTGAC	59	79
	Rev—GTCATCCTCCTCCACTACGG	60	
TEK receptor tyrosine kinase 2	For—TGGAGAAGGACATCCTGGAC	60	99
	Rev—GCTGTCTGGCTTTTGGGTAG	60	
Vascular endothelial growth factor A	For—AATGATGAAGCCCTGGAGTG	60	90
	Rev—TATGTGCTGGCTTTGGTGAG	60	
Vascular endothelial growth factor B	For—AGGAGAGTGCTGTGAAGCCA	61	194
	Rev—TGTGCATCTAGTCTTGAGCTAAGTT	60	
Vascular endothelial growth factor receptor 1	For—CCCATCGGCAGACCAATACA	60	114
	Rev—TATTGAGGTCCGTGGTGACG	59	
Vascular endothelial growth factor receptor 2	For—CTCGTACGGACCGTTAAGC	60	94
	Rev—CTCATCCAAGGGCAGTTCAT	60	

### Statistical analysis

Statistical analysis was performed using Graphpad Prism v7. Corneal vascular area was compared between male and female rats using the Mann-Whitney test. Gene expression data were compared using a two-way ANOVA followed by Tukey post-hoc correction for multiple comparisons. Alpha was set at 0.05.

## Supporting information

S1 Fig Representative images of male and female rat eyes following silver nitrate cautery. Eyes from female **(a,c,e)** and male **(b,d,f)** inbred Sprague-Dawley rats were photographed at days 1, 7 and 14 following silver nitrate cautery of the cornea. The cauterized portion of the cornea was visible the day following cautery in females **(a)** and males **(b)**. Vessels were evident 7 days post cautery in both female **(c)** and male **(d)** eyes. The vessels in the males appeared to infiltrate the cornea more densely. The cornea was oedematous in both males and female rats. Fourteen days post corneal cautery, oedema had resolved in both female **(e)** and male **(f)** eyes, so that the iris vessels were again visible through the cornea. Images were captured using an Olympus E-330 digital camera attached to a Leica Wild M690 ophthalmic surgical microscope.
(TIF)

S2 Fig Representative images of corneal neovasculature in male and female rats 14 days after cautery. (TIF)

S3 Fig Immunohistochemistry for VEGF-R1 in male and female untreated and cauterized corneas. There was no discernable difference in VEGF-R1 expression between untreated female and male corneas. VEGF-R1 expression was observed in the corneal epithelium and endothelium. Cautery resulted in an increase in VEGF-R1 expression in both females as well as males. This increase was most apparent in female rats in the corneal endothelium and was associated with an inflammatory cell infiltrate. Scale bars 50 μm.
(TIF)

S4 Fig Standard curves for primers used for quantitative PCR. A seven point standard curve with dilutions of standard pool cDNA ranging from 1/5 to 1/3645 was generated. A minimum of 5 points were used to perform calculation of the amplification efficiency. The x-axis depicts the log cDNA concentration while the y-axis shows the mean C_T_. Hypoxanthine phosphoribosyltransferase (HPRT) and beta-actin (b-Actin) were used as reference genes.
(TIF)

S1 Table Inflammatory infiltrate in the cornea of male and female rats. H&E stained sections of corneas were assessed by a pathologist masked to the sex of the samples, 72 hours and 14 days post cautery. The inflammatory infiltrate was scored semi-quantitatively. 0 no inflammatory cells, + scant inflammatory cells distributed singly, ++ moderate numbers of inflammatory cells with some groups, +++ marked numbers, some sheets of inflammatory cells.
(TIF)

S2 Table Average normalised expression (± SD) of genes implicated in regulation of angiogeisis compared across male and female Sprague-Dawley rats. The expression in normal and cauterised tissue was compared.
(TIF)
