# Supplementary figures and images for: Sex differences in corneal neovascularization in response to superficial corneal cautery in the rat
Source: PLoS One. 2019 Sep 3;14(9):e0221566. doi: 10.1371/journal.pone.0221566 (PMC6719872; doi:10.1371/journal.pone.0221566)

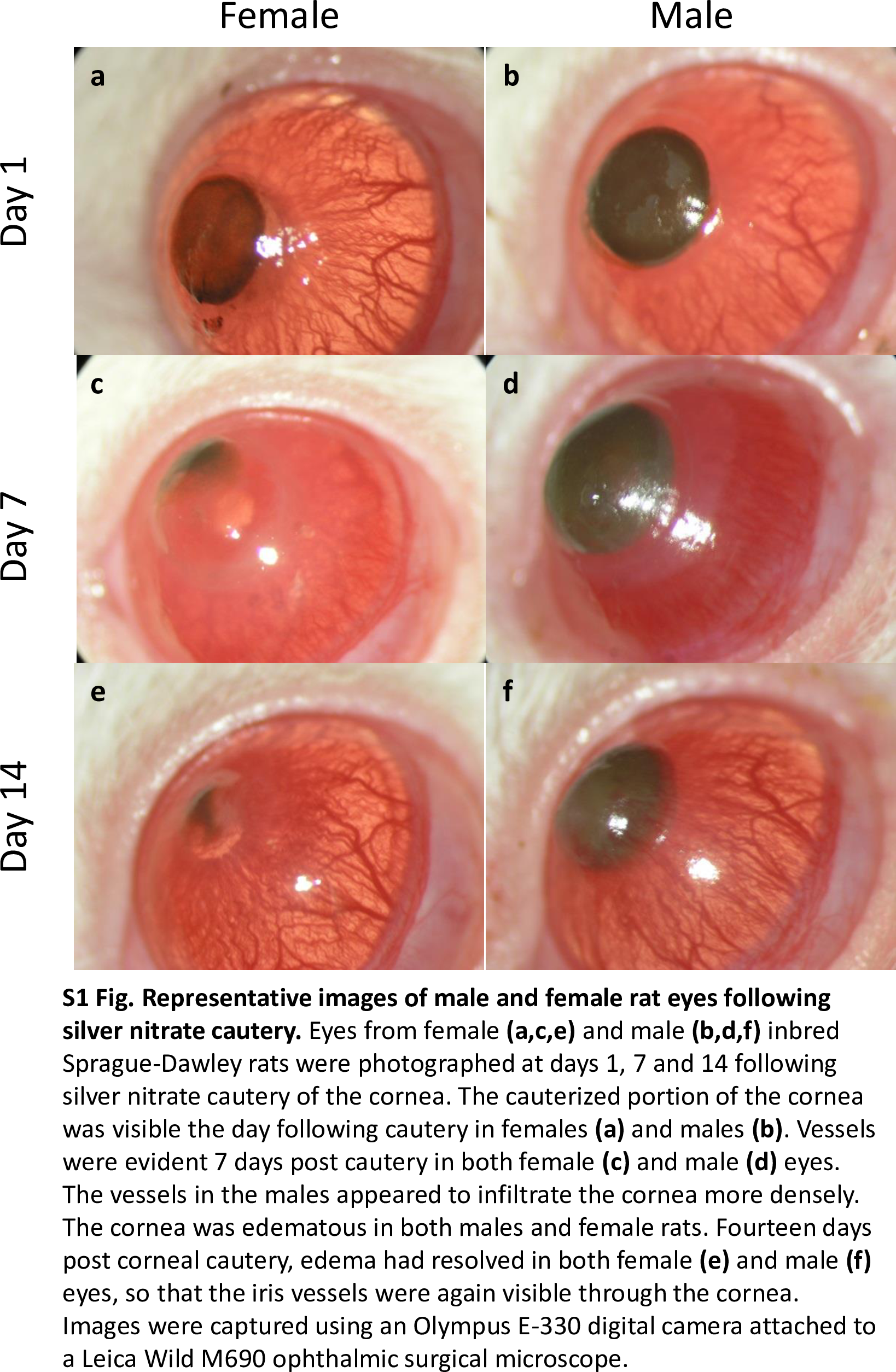

Supplement: S1 Fig — Eyes from female (a,c,e) and male (b,d,f) inbred Sprague-Dawley rats were photographed at days 1, 7 and 14 following silver nitrate cautery of the cornea. The cauterized portion of the cornea was visible the day following cautery in females (a) and males (b). Vessels were evident 7 days post cautery in both female (c) and male (d) eyes. The vessels in the males appeared to infiltrate the cornea more densely. The cornea was oedematous in both males and female rats. Fourteen days post corneal cautery, oedema had resolved in both female (e) and male (f) eyes, so that the iris vessels were again visible through the cornea. Images were captured using an Olympus E-330 digital camera attached to a Leica Wild M690 ophthalmic surgical microscope. (TIF) [file pone.0221566.s001.tif]

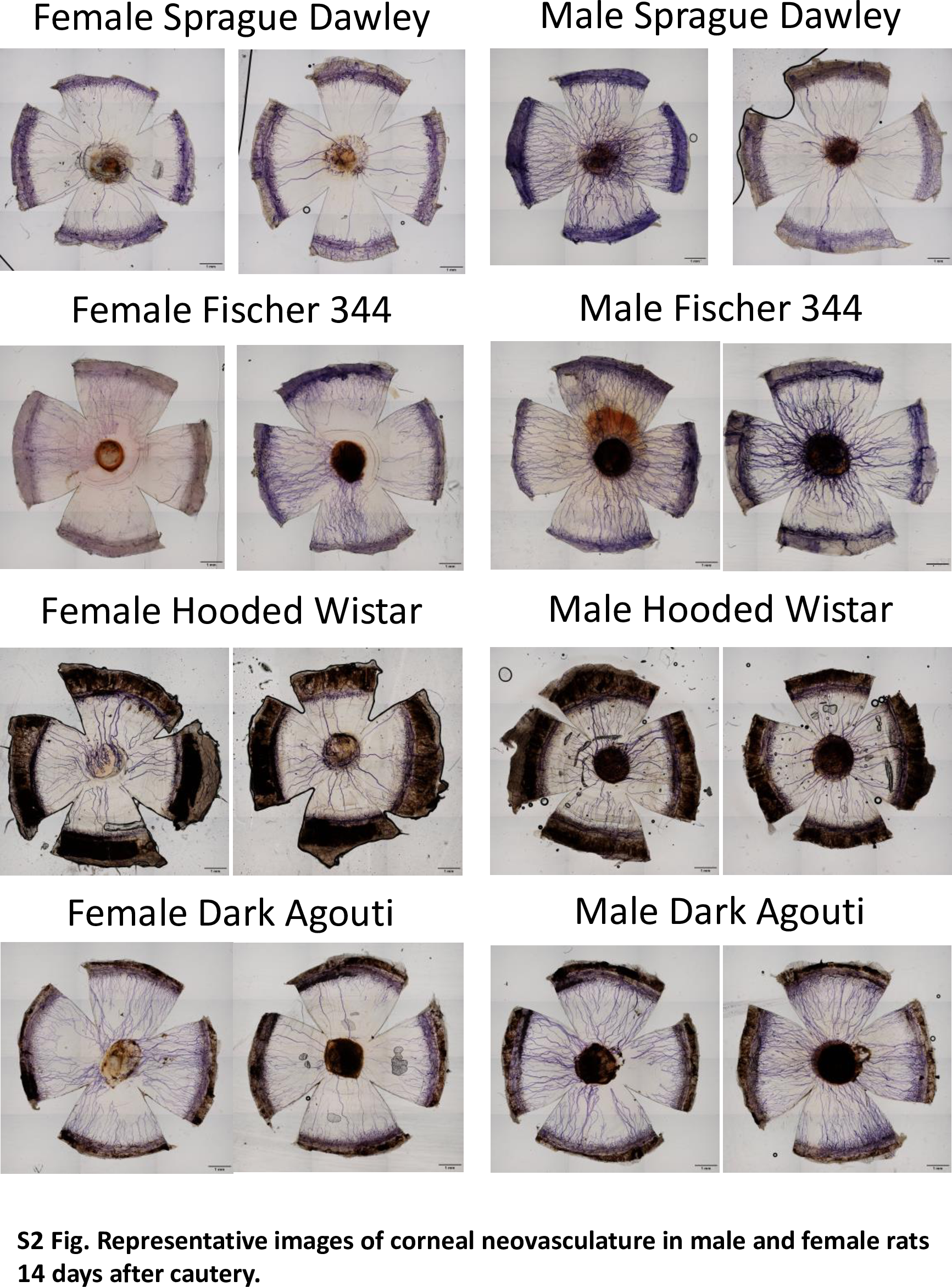

Supplement: S2 Fig — (TIF) [file pone.0221566.s002.tif]

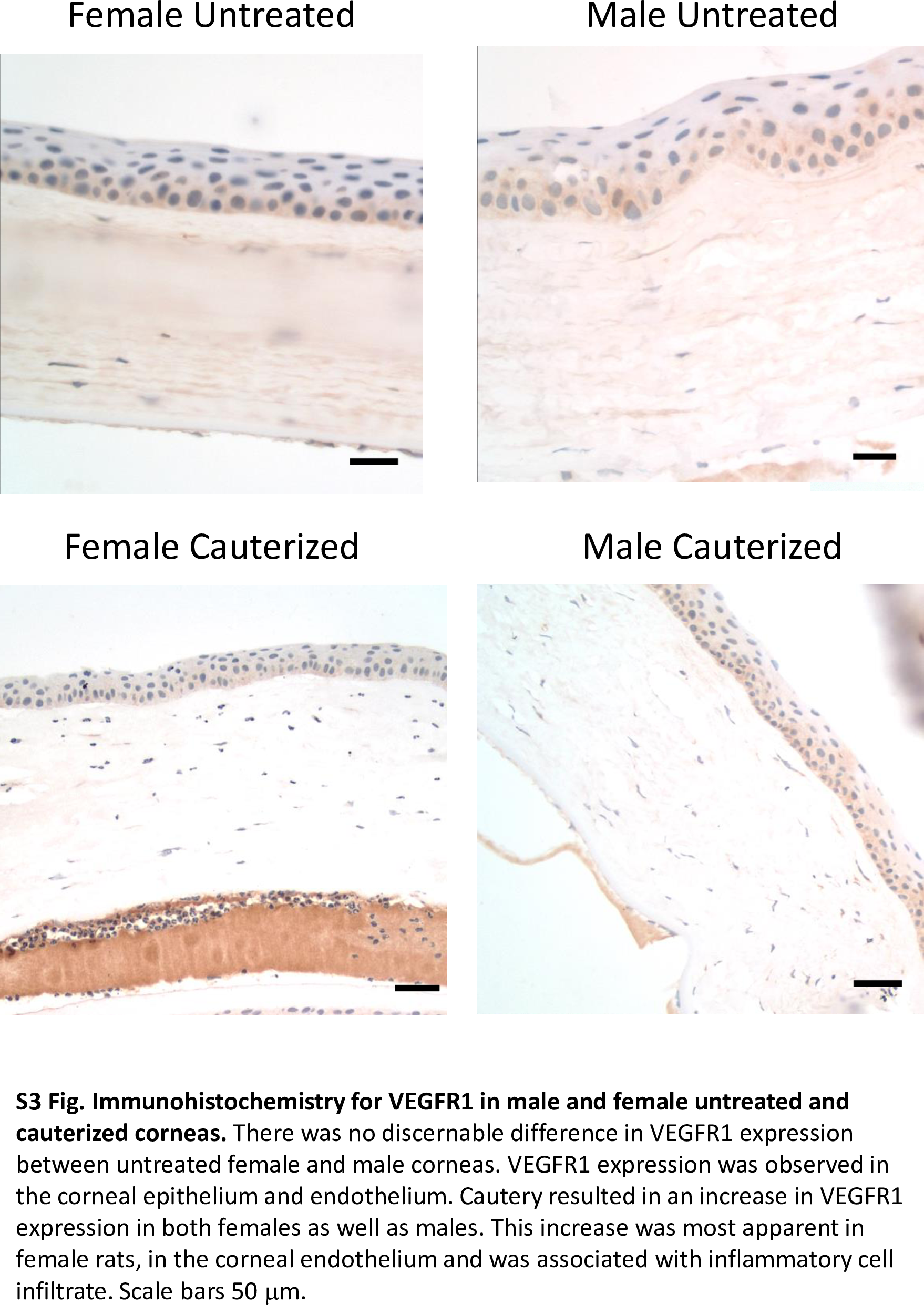

Supplement: S3 Fig — There was no discernable difference in VEGF-R1 expression between untreated female and male corneas. VEGF-R1 expression was observed in the corneal epithelium and endothelium. Cautery resulted in an increase in VEGF-R1 expression in both females as well as males. This increase was most apparent in female rats in the corneal endothelium and was associated with an inflammatory cell infiltrate. Scale bars 50 μm. (TIF) [file pone.0221566.s003.tif]

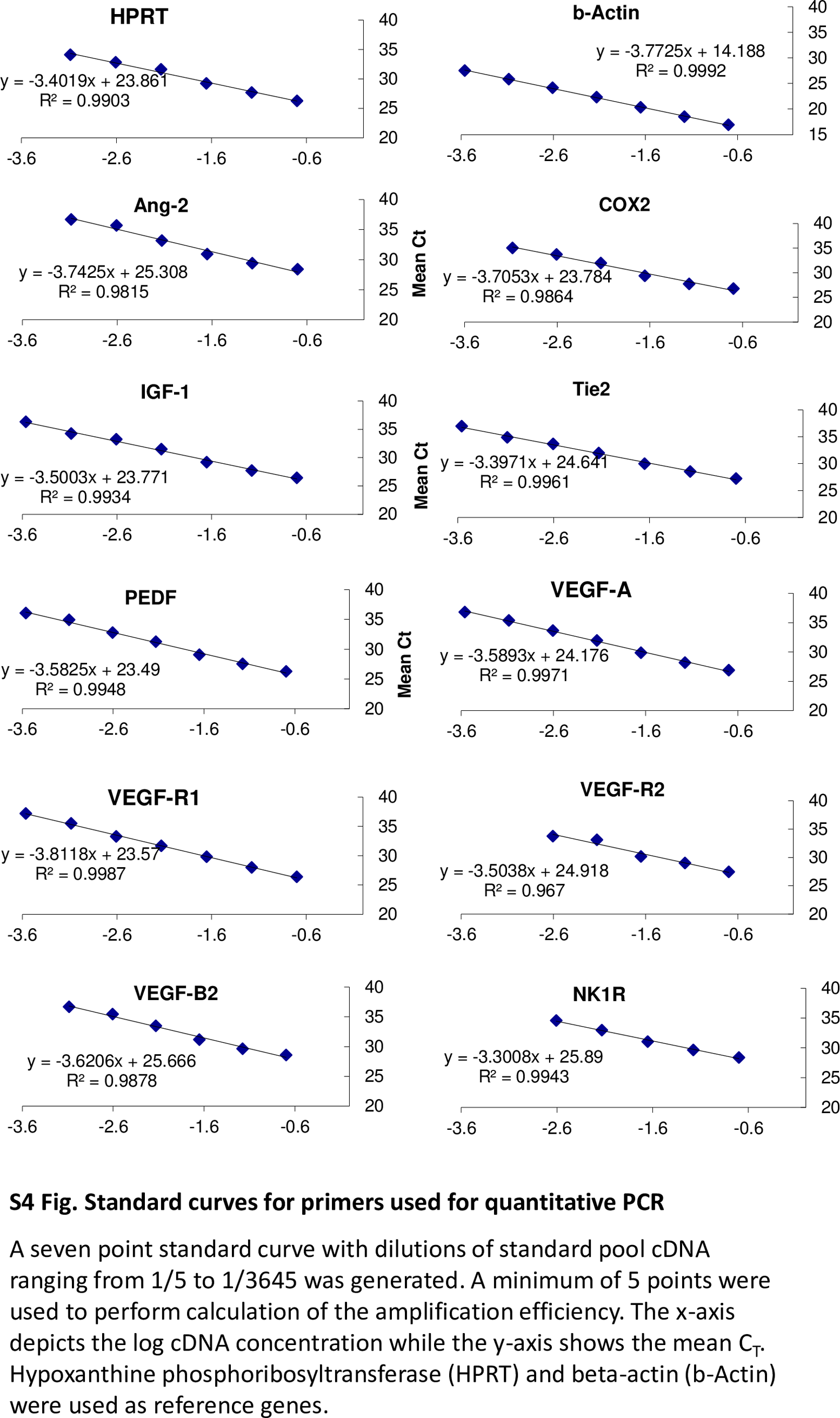

Supplement: S4 Fig — A seven point standard curve with dilutions of standard pool cDNA ranging from 1/5 to 1/3645 was generated. A minimum of 5 points were used to perform calculation of the amplification efficiency. The x-axis depicts the log cDNA concentration while the y-axis shows the mean CT. Hypoxanthine phosphoribosyltransferase (HPRT) and beta-actin (b-Actin) were used as reference genes. (TIF) [file pone.0221566.s004.tif]

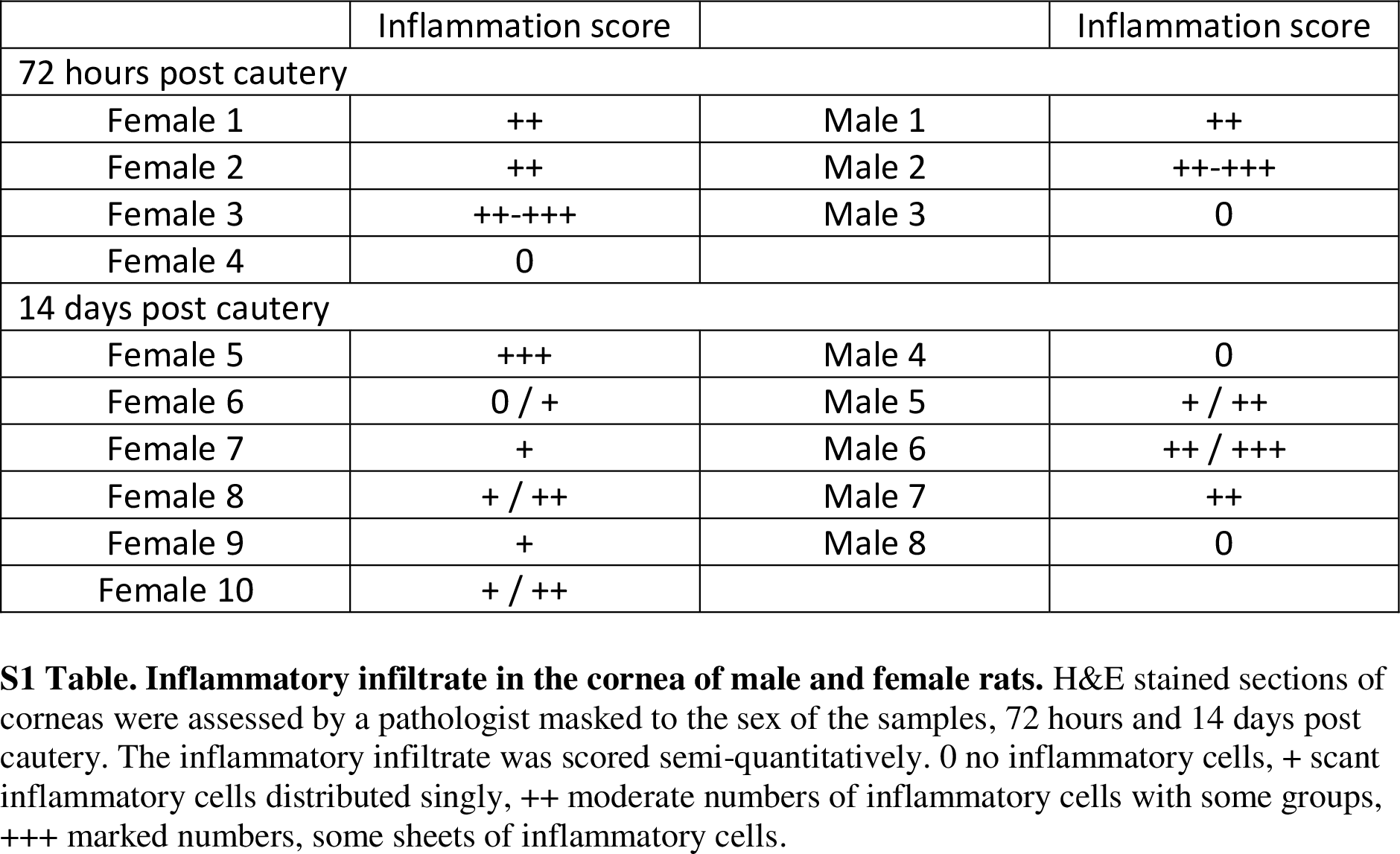

Supplement: S1 Table — H&E stained sections of corneas were assessed by a pathologist masked to the sex of the samples, 72 hours and 14 days post cautery. The inflammatory infiltrate was scored semi-quantitatively. 0 no inflammatory cells, + scant inflammatory cells distributed singly, ++ moderate numbers of inflammatory cells with some groups, +++ marked numbers, some sheets of inflammatory cells. (TIF) [file pone.0221566.s005.tif]

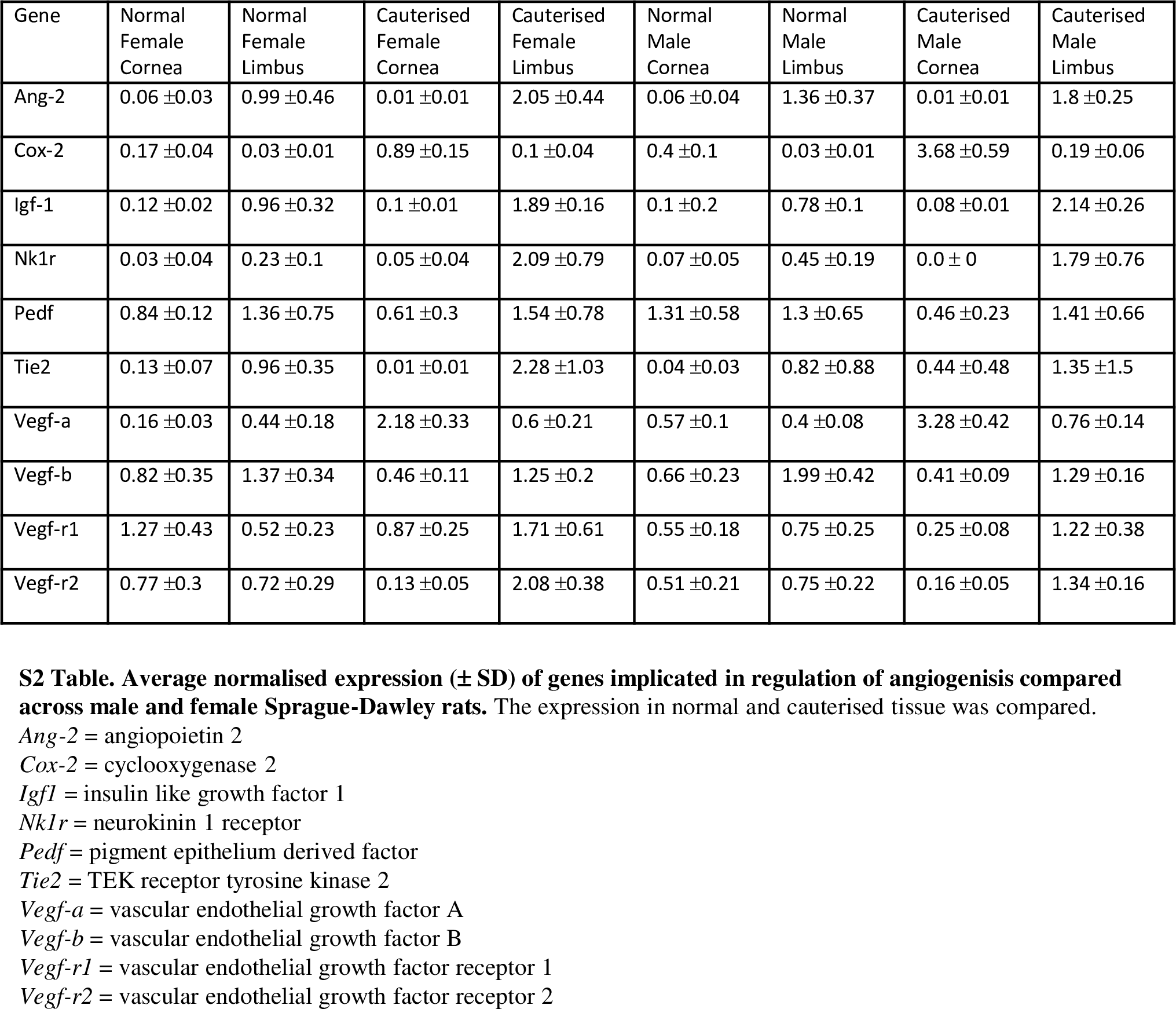

Supplement: S2 Table — The expression in normal and cauterised tissue was compared. (TIF) [file pone.0221566.s006.tif]
